# The effect of concentrated growth factors in the treatment of periodontal intrabony defects

**DOI:** 10.4155/fsoa-2016-0019

**Published:** 2016-09-15

**Authors:** Jing Qiao, Jinyu Duan, Yong Zhang, Yi Chu, Changzhou Sun

**Affiliations:** 1 Department of Periodontology, Outpatient Center, Peking University School & Hospital of Stomatology, Beijing, China

**Keywords:** bovine porous bone mineral, concentrated growth factors, periodontal intrabony defects, periodontal regeneration

## Abstract

**Aim:**

To investigate the effect of concentrated growth factors (CGFs) in human intrabony defect treatment.

**Methods:**

Thirty-one intrabony defects were randomly treated with CGFs + bovine porous bone mineral (BPBM) or BPBM alone. Probing depth, clinical attachment level and hard tissue fill were evaluated at baseline and 1 year post surgery.

**Results:**

No differences in any of the investigated parameters were observed at baseline. At 1 year post therapy, both groups showed significant improvement in clinical parameters (p < 0.001). CGFs + BPBM was more effective than BPBM alone at decreasing probing depth (4.2 ± 1.3 mm vs 3.0 ± 1.6 mm) and clinical attachment level gain (3.7 ± 1.3 mm vs 2.4 ± 1.1 mm; p ≤ 0.05). A favorable increase of hard tissue fill was noted in CGFs + BPBM group compared with BPBM group (p > 0.05). The contents of growth factors in CGFs were statistically higher than those in platelet poor plasma (p < 0.001).

**Conclusion:**

Addition of CGFs significantly improved clinical effectiveness of BPBM for intrabony defect treatment.

## Lay abstract

Concentrated growth factors (CGFs) are a new generation of platelets concentrates, which contain abundant growth factors. We assumed that CGFs might promote periodontal (gum) regeneration. In the present study, we used CGFs in the treatment of periodontal intrabony defects. The results at 1 year post surgery indicated that the addition of CGFs significantly improved the clinical effectiveness of bone graft alone. More research is needed to better understand this effect.

**Figure 1.**
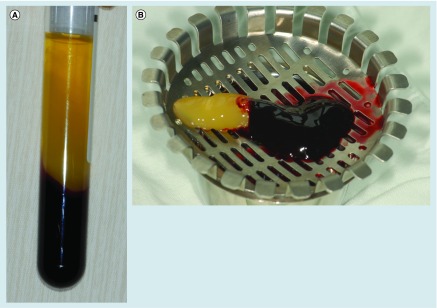
**Concentrated growth factors.** **(A)** Three blood fractions were obtained through centrifuge process (1) a superior phase represented by the serum; (2) an interim phase represented by a very large and dense polymerized fibrin block containing the CGFs, white blood cells and stem cells; and (3) the lower red blood cell layer. **(B)** CGFs separated from platelet-poor plasma.

**Figure 2.**
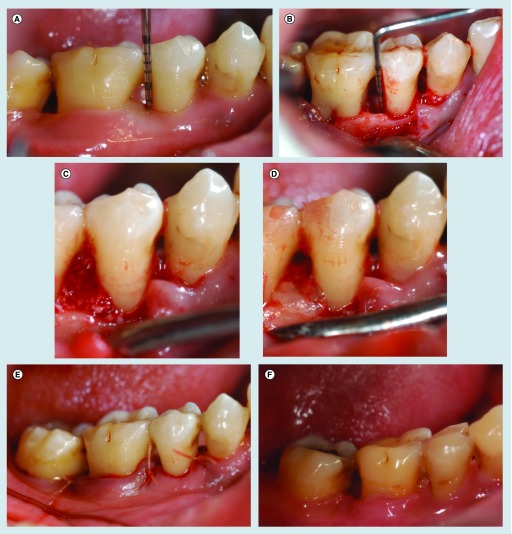
**(A)** An example in the experimental group: the second right mandibular premolar, baseline. **(B)** Intra-surgical findings. **(C)** Mixture of CGFs and BPBM granules was placed into the defect. **(D)** CGFs membrane was placed. **(E)** Flap sutured. **(F)** 1 year post-surgery.

**Figure 3.**
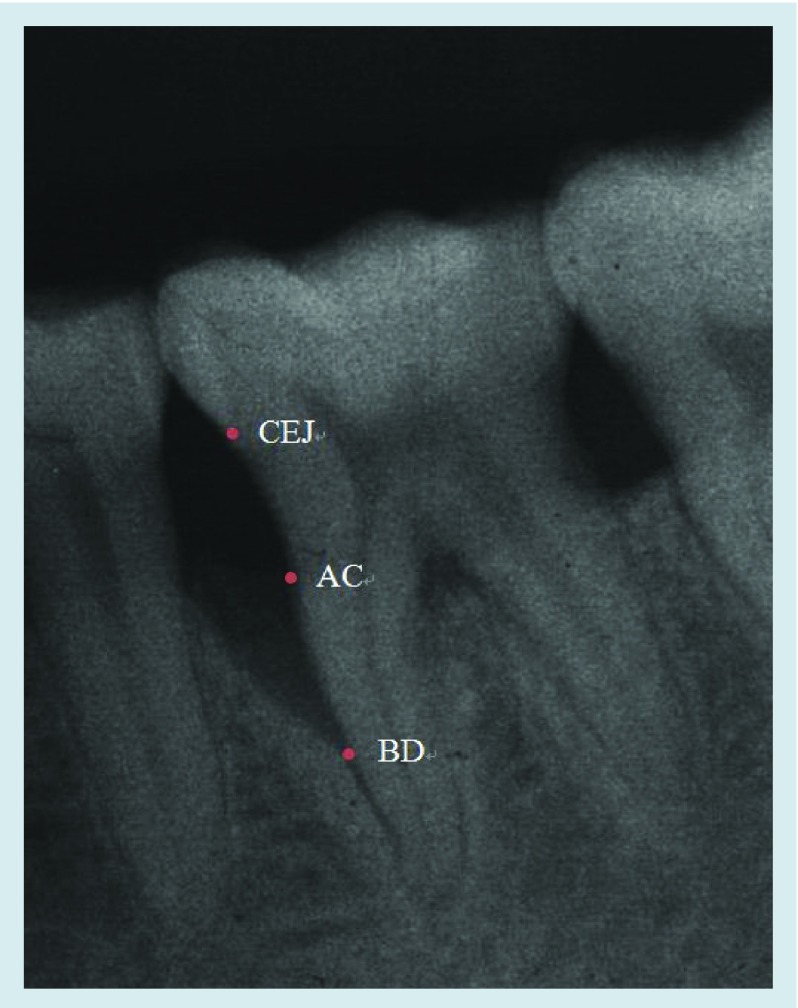
**Radiographic landmarks.** AC: Alveolar crest; BD: Base of the defect; CEJ: Cemento–enamel junction.

**Figure 4.**
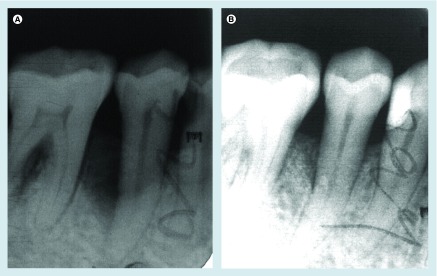
**Periapical radiograph of the second right mandibular premolar 1 year post-surgery.**

Periodontitis is an inflammatory disease that leads to the loss of tooth-supporting tissues. Tissue loss caused by periodontal disease is typically treated by a variety of regenerative treatment modalities, including bone grafts, guided tissue regeneration (GTR) and growth factors, to reform the tooth's supporting tissues [1–5].

However, a common problem of GTR therapy is the high variability and low predictability of healing outcomes, even as complete and predictable reconstruction of periodontal tissues is difficult to obtain with any therapeutic plan [6,7]. Specifically, the degree of healing depends on both the local characteristics of the intrabony defects and the regenerative potential of the residual periodontal tissues [4,8,9].

Polypeptide growth factors are one of the fundamental elements in tissue engineering, which have shown an important role in the growth and differentiation of cells involved in periodontal wound healing [10,11]. Recombinant forms of growth factors may have positive effects in experimental studies but are not practical for clinical application owing to complexity of application and their high costs.

Platelets, a major resource of autogenous growth factors, are among the first cells to reach a wound site and initiate the healing process [12].

Platelet-rich plasma (PRP) was the first generation of platelet gel used in periodontal regeneration therapy [13–18]. While the potential benefits of this procedure have been critiqued, many of the discrepancies are likely more related to the lack of suitable methodological standardization and definition of the different PRP preparations than to any functional inadequacies, as the protocols and biological and surgical techniques used in the administration of the PRPs differ widely between study groups [19,20].

Platelet-rich fibrin (PRF), the second generation of platelet concentrate products, exhibits the same properties as PRP with the advantages of osteogenicity, a simple preparation process and a lack of bovine thrombin and anticoagulants, as it is produced from autologous blood [21–24].

Concentrated growth factors (CGFs) were first developed by Corigliano in 2010 [25]. CGFs are produced by centrifuging blood samples at alternating and controlled speeds using a special centrifuge (Medifuge, Silfradentsrl, Italy). Different centrifugation speeds permit the isolation of a much larger and denser fibrin matrix richer in growth factors than typically found in PRP or PRF. Rodella *et al*. observed a fibrin network constituted by thin and thick fibrillar elements, and multiple platelet cell elements were observed forming a cell aggregate trapped among the fibrin network [26]. Their study demonstrated the presence of TGF-β1 and VEGF in CGF and red blood cell (RBC) layers, suggesting that an improved CGF isolation procedure could optimize the amount of growth factors in the CGF layer. Furthermore, their results showed a high number of CD34-positive cells in CGFs – CD34 having been demonstrated to play an important role in vascular maintenance, neovascularization and angiogenesis [26].

In theory, CGFs appear to exhibit superior potential for tissue regeneration in clinical and biotechnological applications, as evident in a report of sinus and alveolar ridge augmentation [27]; however, there are few studies supporting this. The purpose of this study was to evaluate the effect of CGFs in the treatment of periodontal intrabony defects.

## Materials & methods

### Study population

Seventeen patients (ten females and seven males; aged from 24 to 64 years, average age 47.7 ± 13.9 years) suffering from periodontal disease were included in this study. All patients were informed of the nature of this study and signed an informed consent prior to their inclusion. The study was performed in accordance with the Helsinki Declaration of 1975, as revised in 2000, and the study protocol was reviewed and approved by the university ethical board (Peking University, School and Hospital of Stomatology). All patients were treated by the same experienced surgeon at the Department of Periodontology, in the Outpatient Center, at the School and Hospital of Stomatology, Peking University.

Subject inclusion was based on the presence of at least one tooth with a probing depth (PD) of ≥6 mm and radiographic evidence of intrabony defect of ≥3 mm after initial periodontal therapy. Exclusion criteria consisted of patients with systemic diseases, pregnant and/or lactating women, patients taking any drug known to affect the number or function of platelets in the past 3 months and patients with abnormal platelet counts. Teeth nonresponsive and abnormal-responsive to cold stimulation or teeth endodontically treated were also excluded from this study.

### Study design

All patients underwent initial therapy, consisting of oral hygiene instruction, full-mouth scaling and root planning and occlusal adjustment when indicated. After periodontal re-evaluation, the patients were included in this study.

Six patients had a pair of intrabony defects, seven patients had one defect and four patients had three defects (two of the three were a matched pair). In total, 31 defects were included in this study. For the test group, CGFs mixed with BPBM (bovine porous bone mineral, Bio-Oss, Geistilich, Switzerland) was grafted into the intrabony defect; for the control group, BPBM alone was used. Prior to surgery, each defect was randomly assigned to either the test group (n = 15) or the control group (n = 16) using the randomized block approach. Split-mouth design was used for matched defect pairs; in other words, one defect was assigned according to the random table, and then the matched defect was assigned to the other group. Blood samples were also collected from all patients regardless of CGFs application.

### Intra-examiner reproducibility

Clinical measurements of the included teeth were obtained by the same trained periodontist. Five patients, each showing ten teeth (single- and multirooted) with a PD of ≥6 mm on at least one aspect of each tooth, were used to calibrate the examiner. The examiner evaluated the patients on two separate occasions, 48 h apart. Intra-observer agreement produced a Cohen's kappa (κ) of 0.859.

### Clinical measurements

All baseline clinical parameters were obtained on the day of surgery by the same periodontist, who was also blind to the type of treatment. Final parameters were taken 1 year postoperatively by the same examiner, again blind to the type of treatment. The following measurements recorded prior to surgery and 1 year after surgery were obtained using the same type of periodontal probe (UNC 15, Hu-Friedy, IL, USA): plaque index [28], bleeding index [29], PD, gingival recession (REC) and clinical attachment level (CAL). The cemento–enamel junction (CEJ) was used as the reference point.

### CGF preparation

CGFs were produced as follow: 9 ml of blood was drawn in sterile Vacuette tubes (Greiner Bio-One, GmbH, Kremsmunster, Austria) without anticoagulant solutions. These tubes were then immediately centrifuged (Medifuge, Silfradentsrl, Sofia, Italy) using a program with the following characteristics: 30 s acceleration, 2 min at 2700 rpm, 4 min at 2400 rpm, 4 min at 2700 rpm, 3 min at 3000 rpm and 36 s deceleration and stop. At the end of the process, three blood fractions were created (Figure 1A): a superior phase represented by the serum (blood plasma without fibrinogen and coagulation factors, platelet poor plasma, PPP); an interim phase represented by a very large and dense polymerized fibrin block containing the CGFs, white blood cells and stem cells; and the lower RBC layer [26,30].

When used in surgery, the fibrin block and RBC layer beneath it were cut into pieces of 1∼2 mm, and mixed with BPBM granules (particle size 0.25–1.0 mm, BioOss, Geistlich, Wolhusen, Switzerland) at a relative volume of 1:1. This was all mixed and homogenized mechanically for ∼6 s using the Silfradent Round Up mixer (Silfradent, Italy) to obtain a more uniform mixture. Another part of the fibrin block separated from the red phase was made into a shaped membrane using forceps, and the CGFs membrane was also prepared.

### Evaluation of contents of growth factors in CGFs

Another 9 ml of blood was drawn from the same patient at the same time, and another piece of CGFs was prepared using the method mentioned above. The PPP layer was aspirated and stored at -20°C. The fibrin block and RBC layer beneath it were separated and cut into pieces of 1∼2 mm. Then the mixture was centrifuged at 4500 rpm under 4°C for 15 min. The supernatant was aspirated and stored at -20°C for measurement of growth factors in it.

The levels of following growth factors in PPP and CGFs were evaluated using double antibody sandwich ELISA: PDGF-BB, TGF-β1, IGF-1, VEGF. OD values were measured under 450 nm wavelength absorbance using microplate reader (Bio-Rad, USA).

### Surgical procedure & intrasurgical measurements

The same surgeon performed all operations under local anesthesia (4% articaine with epinephrine 1:100,000). Buccal and lingual sulcular incisions were made, and mucoperiosteal flaps were elevated. Thorough debridement of the defects was achieved with hand instruments. The surgical area was irrigated with copious amounts of sterile saline. During surgery, distance from the CEJ to the most coronal extension of the alveolar bone crest (CEJ-AC) and distance from the CEJ to the base of the defect (CEJ-BD) were measured using a UNC-15 periodontal probe (Hu-Friedy, USA). Depth of the intrabony defects (AC-BD) was defined as (CEJ-AC)-(CEJ-BD).

During surgery, the prepared mixture of CGFs and BPBM granules was placed into the defects in the CGFs + BPBM group; the mixture was sticky and easy to handle. Following grafting, the CGFs membrane was trimmed and adapted over the entire defect so as to cover 2–3 mm of the surrounding alveolar bone and to ensure stability of the wound and of the graft material. For control sites, BPBM grafts alone were used. After grafting, the flap was repositioned to accomplish complete interproximal closure. If the closure could not be achieved by the repositioned flap, the coronal reposition flap would be used. The flap was then sutured with 4–0 absorbable sutures with single interrupted sutures and modified vertical mattress, if necessary.

Figure 2 showed No. 4 case in the CGF + BPBM group.

### Postoperative care

All patients were prescribed to take 500 mg amoxicillin three-times a day for 1 week and instructed to rinse with 0.12% chlorhexidine twice a day for 1 month. Sutures were removed 14 days after the surgery. Patients received professional prophylaxis every 2 weeks during the first 2 months and were followed up with at 3, 4, 5, 6, 9 and 12 months after surgery.

### Radiographic measurements

Periapical radiographs were taken with standardized projection geometry at baseline and 1 year after surgery. All radiographs were digitized using a scanner with a transparency module (Hewlett-Packard Scanjet XPA 7400c, Avision, China) to a resolution of 256 pixels with 8 bits of gray-level resolution per pixel and saved as TIFF files. The images were viewed by the same trained radiographer on a 19″ Viewsonic VA703B monitor (Viewsonic, CA, USA) set at a screen resolution of 1280 × 1024 pixels. No time restriction was placed on the observer, and images were viewed at 1-week intervals. The radiographer was blinded to the treatment. When evaluating the images, the room lights were turned off. Measurements were made to the nearest 0.5 mm using Photoshop software with a linear measurement tool and a digital magnifying lens. Before the study, the radiographer was calibrated. Intra-observer agreement produced a Cohen's kappa of 0.891.

The following measurements were performed on both preoperative and postoperative radiographs: distance between the CEJ and alveolar crest (CEJ-AC), distance between the CEJ and base of the defect (CEJ-BD), and the depth of the intrabony defects – distance between alveolar crest and base of the defect (AC-BD) (Figure 3).

Figure 4 showed the periapical radiographic image of the No. 4 case in the CGF + BPBM group at baseline and 1-year post surgery.

### Statistical analysis

A power analysis determined that a sample size of 30 defects would be sufficient to detect a difference of 5% or greater between CGFs + BPBM group and BPBM alone. Results were averaged (mean ± standard deviation [SD]) for each parameter. SPSS 13.0 (SPSS, IL, USA) was used to analyze the data. Comparisons of baseline clinical and radiographic measurements and of changes from baseline to 1 year between the two treatment groups were performed using paired t-tests. The levels of growth factors in PPP and CGFs were compared using paired t-tests. A p-value of ≤0.05 was considered significant for all statistical analyses.

## Results

All sites healed uneventfully without flap dehiscence or infection. No complications such as abscesses or infections were observed throughout the study period.

### Baseline defect comparability

No differences were observed in the gender distribution between the groups (four females and four males in the CGFs + BPBM group and five females and four males in the BPBM group). The defects displayed a comparable distribution and type in the two groups (Table 1). Intrabony defect depths, as measured during surgery, in the CGFs + BPBM and BPBM groups were not significantly different (4.6 ± 1.8 mm vs 5.0 ± 1.4 mm, respectively) (Table 2).

### Comparison of clinical measurements at baseline & 1 year post surgery

Plaque index and bleeding index at baseline and 1 year post surgery were not significantly different between the CGFs + BPBM and BPBM groups (Table 3).

Mean PD, REC and CAL at baseline in the CGFs + BPBM and BPBM groups were not significantly different (p > 0.05) (Table 4). Mean PD at baseline was 7.3 ± 1.5 mm in the CGFs + BPBM group and 7.5 ± 1.2 mm in the BPBM group (p > 0.05); however, mean PD at 1 year decreased signiﬁcantly in both groups (decrease of 4.2 ± 1.3 mm and 3.0 ± 1.6 mm, respectively) compared with the baseline data (p < 0.001), and the decrease of mean PD in the CGFs + BPBM group was significantly greater than that of the BPBM group (p < 0.05) (Table 4).

Mean REC at baseline in the CGFs + BPBM and BPBM groups was not signiﬁcantly different (p > 0.05) (Table 4); however, mean REC at1 year increased significantly in both groups (increase of 1.7 ± 1.5 mm and 1.8 ± 1.1 mm, respectively) compared with the baseline data (p < 0.01), but neither group exhibited an increase significantly different than the other (p > 0.05).

Mean CAL at baseline in the CGFs + BPBM and BPBM groups was not signiﬁcantly different (p > 0.05) (Table 4); however, mean CAL at 1 year increased significantly in the CGFs + BPBM group (3.7 ± 1.3 mm) (p < 0.05) but not in the BPBM group (2.4 ± 1.1 mm) (p > 0.05). Notably, a 3-mm threshold change was chosen as a cutoff by which to judge a significant clinical improvement. A CAL change of ≥3 mm (significant) occurred in 11 cases (73.3%) in the CGFs + BPBM group (Table 5) and in only six cases (37.5%) in the BPBM group (Table 5).

### Comparison of radiographic measurements at baseline & 1 year post surgery

There was no statistically significant difference in radiographic measurements between the two groups prior to treatment (p > 0.05). Table 6 presents the baseline and 1 year radiographic measurements of the two groups. The mean crestal bone resorption (CEJ-AC) showed significant differences between baseline and 1 year for both groups (p < 0.001). Additionally, significant hard tissue fill (CEJ-BD reduction) occurred in both groups 1 year after treatment (p < 0.001). The CGFs + BPBM group presented more favorable gains in radiographic parameters (3.8 ± 1.5 mm) than the BPBM group (2.8 ± 1.4 mm); however, the difference between the two groups was not significant (p > 0.05).

### Comparison of contents of growth factors in CGFs & PPP

The average levels of various growth factors in CGFs were significantly higher than that in PPP (p < 0.001) (Table 7).

## Discussion

This study compared combination CGFs + BPBM with BPBM alone in the treatment of human intrabony defects. The results indicate that CGFs + BPBM treatment was effective in significantly improving clinical and radiographic parameters at 1 year after surgery compared with BPBM alone. However, although significantly different from baseline values, CAL gain and hard tissue fill recorded for the BPBM alone therapy group is limited.

### Clinical results

Few studies exist on the effect of CGFs in the treatment of periodontal intrabony defects. Therefore, a comparison of the data from this study on CGFs + BPBM treatment can only be compared with that of other studies on the use of PRP in the treatment of periodontal intrabony defects.

The improvement in the selected clinical parameters was similar to most previously reported studies. In 2006, we evaluated the effectiveness of PRP as an adjunct to BPBM in the treatment of human intrabony defects and found a statistically significant improvement in the PRP + BPBM group compared with the BPBM group, with a PD reduction of 4.78 mm and a CAL gain of 4.52 mm [31]. Lekovic *et al*. compared the clinical effectiveness of two regenerative techniques (PRP + GTR + BPBM vs PRP + BPBM) for intrabony defects in humans [16]. At 6 months post surgery, both treatments showed significant improvement compared with the baseline values with no statistically significant difference. For the PRP + BPBM group, PD reduction was 3.98 mm on buccal sites and 3.94 mm on lingual sites, and CAL gain was 3.78 mm on buccal sites and 3.84 mm on lingual sites. In a 6 month clinical trial, Hanna *et al*. compared the clinical outcomes obtained by PRP + BPBM to those obtained from BPBM alone in the treatment of periodontal intrabony defects. They reported 3.54 mm of PD reduction and 3.15 mm of CAL gain in the PRP + BPBM group, each significantly greater than that of the BPBM group [17]. Parimala *et al*. compared the 9 months efficacy of BPBM with and without PRP for the treatment of 28 human periodontal intrabony defects, noting a PD reduction of 6.60 mm for the PRP + BPBM-treated sites and a CAL gain of 4.70 mm for the PRP + BPBM group [32]; their results appeared to be more favorable than those of other studies.

A 3 mm threshold change was chosen as a cutoff by which to judge a significant clinical improvement. A CAL change of ≥3 mm occurred in 73.3% of patients in the CGFs + BPBM group in the present study, while Hanna *et al*. previously noted CAL changes of ≥ 3 mm 76.9% (ten cases) of patients in their PRP + BPBM group.

### Hard tissue fill

In this study, the average radiographic hard tissue fill is in accordance with previous reports on the clinical benefits of a regenerative approach employing PRP combined with BPBM.

Parimala *et al*. reported a significant radiographic defect hard tissue fill of 4.04 mm in the PRP + BPBM group [32]. In our 2006 study, bone probing level (BPL), measured with a UNC-15 probe under local anesthesia, was used to determine hard tissue fill; BPL reduction was 4.56 mm in the PRP + BPBM group [31]. See comment in PubMed Commons below, Lekovic *et al*. evaluated defect hard tissue fill by re-entry surgery at 6 months after regenerative surgery and revealed a reduction of 4.82 mm on buccal sites and of 4.74 mm on lingual sites in the PRP + BPBM group [16]. Notably, each study characterized intrabony defects differently and evaluated bone level using different methods (radiography, bone probing and re-entry surgery). Projection geometry may have also been different. Therefore, a more accurate, standardized method to evaluate defect hard tissue fill in regenerative treatment is needed.

### Possible CGFs mechanism of action

In the present study, inclusion of CGFs improved the outcome of regenerative periodontal intrabony defect treatment. CGFs activity in this respect may be due to its biologic constituents, in addition to chemotactic and mitogenic PDGF, that are involved in tissue regeneration.

Use of different centrifugation speeds permits the collection of abundant growth factors located just below the buffy coat and above the dense clot portion. The results of the present study showed that the levels of TGF-β1, PDGF-BB, IGF-1 and VEGF in CGFs were significantly higher than those in PPP (p < 0.001). Rodella *et al*. confirmed the presence of TGF-β1 and VEGF in CGFs and demonstrated a similar pattern of expression in the RBC layer [26]. Polypeptide growth factors have been shown to play an important role in the growth and differentiation of cells involved in periodontal wound healing [10,11,33]. Unlike PRP, CGFs do not dissolve rapidly following application. Instead, the strong fibrin gel in the matrix addition is slowly remodeled in a similar manner to a natural blood clot. Thus, CGFs prolong the duration of growth factor activity, which is conducive for growth factor synergy, and enhances cell proliferation and osteogenic differentiation [30].

Another likely favorable component in CGFs are stem cells. Using immunohistochemistry, Rodella *et al*. found a higher number of CD34-positive cells in CGF layers than in RBC layers, possibly trapped by the CGF network composition [26]. PRF are known to provide a supportive matrix for circulating mesenchymal stem cells, which are recruited from blood to injured tissue by signaling molecules released from platelets [34]. Moreover, increasing evidence points to the role of circulating CD34-positive cells in vascular maintenance, neovascularization and angiogenesis [35–37].

The fibrin buffy coat is a major component in CGFs. In the present study, the fibrin block is cut into pieces of approximately 1–2 mm, mixed with BPBM and put into the bone defects. It is well known that in periodontal regeneration, unimpeded absorption, adhesion and maturation of the fibrin clot for formation of a connective tissue attachment over a long junctional epithelium is vitally important [38]. The CGFs not only improve the wound stability, which is essential for the establishment of a new connective tissue attachment to a root surface, but also provide a scaffold supporting cytokine attachment and cellular migration. Through the polymerization of the fibrinogen molecules, the fibrin block comprises a 3D polymer network of interwoven fibers. Upon scanning electron microscopic analysis of the fibrin block, Rodella *et al*. observed a fibrin network constituted by thin and thick fibrillar elements, including multiple trapped platelets. A 3D environment is crucial for cell–cell and protein–protein interactions to create tissue symmetry [26].

To date, there is only one published study regarding the effect of CGFs on periodontal ligament stem cells (PDLSCs) *in vitro*. Yu B *et al*. investigated the proliferation and differentiation of beagle PDLSCs cocultured with CGFs and showed that CGFs significantly promoted the proliferation of PDLSCs and exhibited a dose-dependent effect on the activation and differentiation of the stem cells [30]. These data appear to support the hypothesis in the current study; however, future studies are needed to better characterize the mechanism underlying CGFs activity and to support its clinical application.

### The limitation of the present study

There were 31 defects in 17 patients included in this study. Seven patients had only one intrabony defect, which cannot be used in a split-mouth design. A larger sample size and a more rigorous overall experimental design are necessary for future studies. The methods we used to evaluate the regenerative effect included clinical examination and periapical radiographs. Although a good intra-examiner reproducibility was obtained, the re-entry measurement is still the ‘gold standard’ to reflect actual bone change; and periapical radiography is inherently limited in its ability to evaluate true bone level. Furthermore, the suture technique of the surgeon could also affect the outcome of the treatment.

## Conclusion

In conclusion, the results of this study indicate that the CGFs + BPBM combination improved clinical outcome better than BPBM alone in the treatment of human intrabony defects. This also appears the case for hard tissue fill, CAL gain and PD reduction. The CGFs + BPBM group presented more favorable, but not statistically significant, gains in radiographic hard tissue fill. Long-term, multicenter randomized, controlled clinical trials will be required to better understand the clinical and radiographic effects of CGFs on periodontal regeneration.

**Table 1.** **Distribution and type of treated intrabony defects.**

	**CGF + BPBM**	**BPBM**
Maxilla	7	9
Mandible	8	7
Anterior teeth	3	3
Premolars	3	6
Molars	9	7
2 wall	3	4
2–3 wall	6	6
3 wall	6	6

BPBM: Bovine porous bone mineral; CGF: Concentrated growth factors.

**Table 2.** **Mean intrabony defect characteristics in surgery.**

**Treatment**	**CEJ-BD (mm)**	**CEJ-AC (mm)**	**AC-BD (mm)**
CGF + BPBM (n = 15)	9.1 ± 1.1	4.5 ± 1.6	4.6 ± 1.8
BPBM (n = 16)	8.6 ± 1.5	3.6 ± 1.4	5.0 ± 1.4

AC: Alveolar bone crest; AC-BD: Depth of intrabony defect; BD: Base of defect; CEJ: Cemento–enamel junction.

**Table 3.** **Mean plaque index and bleeding index at baseline and 1 year post surgery.**

	**CGFs + BPBM**	**BPBM**
**Plaque index**		
Baseline	0.7 ± 0.3	0.8 ± 0.4
1 year post surgery	0.7 ± 0.2	0.8 ± 0.5
**Bleeding index**		
Baseline	0.9 ± 0.6	0.9 ± 0.5
1 year post surgery	1.0 ± 0.7	1.0 ± 0.7

BPBM: Bovine porous bone mineral; CGF: Concentrated growth factor.

**Table 4.** **Clinical parameters at baseline and 1 year post surgery.**

	**CGFs + BPBM (n = 15)**	**BPBM (n = 16)**	**p-value**
**Probing depth (mm)**			
Baseline	7.3 ± 1.5	7.5 ± 1.2	0.617
1 year post surgery	3.1 ± 0.7	4.5 ± 0.8	0.001
Difference	4.2 ± 1.3	3.0 ± 1.6	0.016
p-value	< 0.001	< 0.001	
**Gingival recession (mm)**			
Baseline	1.3 ± 1.2	1.2 ± 0.9	1.000
1 year post surgery	1.7 ± 1.5	1.8 ± 1.1	0.847
Difference	0.5 ± 0.6	0.7 ± 0.5	0.595
p-value	0.006	< 0.001	
**Clinical attachment level (mm)**			
Baseline	8.5 ± 2.3	8.7 ± 1.9	0.767
1 year post surgery	4.8 ± 2.0	6.3 ± 1.6	0.041
Difference	3.7 ± 1.3	2.4 ± 1.1	0.013
p-value	< 0.001	< 0.001	

BPBM: Bovine porous bone mineral; CGF: Concentrated growth factor.

**Table 5.** **Number of sites and percentage of clinical attachment level gain ≥3 mm in test and control groups.**

**CAL gain ≥3 mm**	**CGFs + BPBM (n = 15)**	**BPBM (n = 16)**
Sites	11	6
Percentage	73.3%	37.5%

BPBM: Bovine porous bone mineral; CAL: Clinical attachment level; CGF: Concentrated growth factor.

**Table 6.** **Mean radiographic measurements of test and control groups.**

	**CGF + BPBM (n = 15)**	**BPBM (n = 16)**	**p-value**
**CEJ-AC (mm)**			
Baseline	4.5 ± 1.4	3.6 ± 1.5	0.245
1 year post surgery	4.9 ± 1.6	4.4 ± 1.5	0.421
Difference	0.5 ± 0.3	0.8 ± 0.5	0.222
p-value	< 0.001	< 0.001	
**CEJ-BD (mm)**			
Baseline	9.3 ± 1.1	8.4 ± 1.9	0.234
1 year post surgery	6.1 ± 1.3	6.4 ± 1.6	0.580
Difference	3.3 ± 1.5	2.1 ± 1.5	0.087
p-value	< 0.001	< 0.001	
**AC-BD (mm)**			
Baseline	4.9 ± 1.8	4.8 ± 1.8	0.935
1 year post surgery	1.1 ± 1.6	1.9 ± 0.6	0.002
Difference	3.8 ± 1.5	2.8 ± 1.4	0.153
p-value	< 0.001	< 0.001	

AC-BD: Depth of intrabony defect; BPBM: Bovine porous bone mineral; CEJ-AC: Distance between CEJ and alveolar crest; CEJ-BD: Distance between CEJ and base of defect; CGF: Concentrated growth factor.

**Table 7.** **The levels of growth factors in platelet poor plasma and concentrated growth factors.**

	**PPP**	**CGFs**	**p-value**
PDGF-BB (ng/ml)	37.58 ± 12.12	176.88 ± 52.32	< 0.001
TGF-β1 (ng/ml)	65.34 ± 21.21	703.02 ± 86.77	< 0.001
IGF-1 (ng/ml)	73.02 ± 18.37	533.69 ± 67.35	< 0.001
VEGF (pg/ml)	44.77 ± 10.05	231.36 ± 44.01	< 0.001

CGF: Concentrated growth factor; PPP: Platelet poor plasma.

**Executive summary**

Addition of concentrated growth factors significantly improved the clinical effectiveness of bovine porous bone mineral to treat intrabony defects.
Long-term, multicenter randomized, controlled clinical trials will be required.
